# Notes and News

**Published:** 1897-08-21

**Authors:** 


### NOTES AND NEWS.

(COLLECTED AND COMMUNICATED.)

A PROPOSAL has been made in Berlin which we do not think would meet with much favour with the medical staff of the London hospitals. This is to establish a central office in connection with the hospitals, where information could be obtained each day of the vacant beds. In respect to epidemics the system would in any case prove invaluable.

THE president of the board of governors of the Royal Bath Hospital and Rawson Convalescent Home (the Hon. A. E. Butler) offered the sum of £5,000 to the convalescent home as far back as January of the present year, subject to certain conditions, that his colleagues could not see their way to accept. However, at a recent meeting, a compromise was effected. Clause I. was altered to read "That a substantial reduction be made in the weekly charge payable by each home patient," instead of "that a uniform reduction of 10s. 6d. for three weeks' stay be made to each home patient," and the word "unanimous" was dropped in Clause III., which provides that the rules of the scheme of accounts be not altered without the approval of the home trustees. The gift is now accepted with thanks.

SERIOUS confusion has overtaken Mr. Hooley's offer to make a donation to the General Hospital, Nottingham, subject to certain conditions. The committee maintain that the only stipulation was that a sum of £10,000 should be collected by working men or others. Mr. Hooley, who had paid £2,000 towards the promised sum, declines to send the remainder, as he states that the stipulation was that £10,000 should be subscribed—not collected—and also that the money was to come from working men. Mr. Hooley offered to submit to arbitration, but this the committee of the collection declined, but invited Mr. Hooley to a meeting, where the matter might be fully discussed. Mr. Hooley did not appear, and the matter now stands that a collection of £10,000 towards the required £20,000 has been made, and Mr. Hooley has made a donation of £2,000, but declines to give the remaining £8,000 expected.

THE Mayor of West Ham has collected £200 for the Prince of Wales's Hospital Fund.

THE funds collected by the Hospital Saturday organisation in New South Wales during the past year amounted to £3,888, and this sum was distributed amongst 15 institutions.

THE fourth Congress for studying tuberculosis will take place in Paris in July, 1898. The discussions will include "The Value of Sanatoria as Prophylactics and as a means of treating tuberculosis, and the use of the X-rays in diagnosing and treating the complaint."

THE foundation-stone of that much-contested institution, the joint infectious hospital for Richmond and Heston-Isleworth, has been laid by the Duchess of Teck. It will contain 46 patients, provision for extension being provided.

THE foundation-stone of a cottage hospital for Ashby, near Leicester, has been laid by Earl Ferrers. It is to contain two wards, for four patients in each, together with operating-room and necessary offices. The cost is estimated at £1,200.

LAST week the Duke of Connaught opened the new hospital at Aldershot. The institution has been built at an expenditure of £3,000. The architecture is simple, and in seventeenth century style. The furniture is oak. The linen has been contributed by the women of Aldershot as a memorial of the Queen's reign. The Duke expressed himself much pleased with all the arrangements. After the ceremony the matron and nurses were presented to him.

AN interesting case has just been tried before Mr. Justice North. Baroness Forester directed her trustees by will to spend £2,000 on a site near Wenlock for a cottage hospital, and a further sum, not exceeding £10,000, in erecting the building and furnishing it; also to expend £5,000 on a site for a convalescent home in the same neighbourhood, and a sum of not more than £30,000 in erecting and furnishing it. Any surplus beyond the £47,000 was to go towards the endowment fund. The estate proved more valuable than the testatrix had thought, and the question had arisen to whom should the surplus go. In fact, did her will cover its disposal or not? Mr. Justice North held that it did, therefore the money belonged to the charity, and he directed a scheme to be settled for dealing with it.

THE following is a list of surgeons on probation of the medical staff of the British Army who were successful at both the London and Netley examinations, arranged in order of merit: S. L. Cummins, J. McArdle, C. H. Hopkins, L. J. C. Hearn, P. Mackessack, J. McD. McCarthy, E. Brodribb, J. Poe, H. L. W. Norrington, H. G. F. Stallard, R. D. Jephson, J. Crean, and A. W. N. Bowen. Mr. Cummins gained the de Chaumont Prize in hygiene, and Mr. Hopkins gained the second Montefiore Prize.

THE following is a list of surgeons on probation of the Indian Medical Service who were successful at both the London and Netley examinations: J. G. P. Murray, S. Anderson, F. H. G. Hutchinson, J. L. Marjoribanks, A. Fenton, J. A. Dredge, and R. W. Knox. The first four gentlemen gained prizes in various subjects.

